# Non-affinity platform for processing knob-into-hole bispecific antibody

**DOI:** 10.1186/s40643-024-00827-8

**Published:** 2024-12-18

**Authors:** Xiaoyang Wang, Min Li, Mengting Li, Huoyan Hong, Kai Gao, Puya Zhao

**Affiliations:** Shanghai AsymBio Biotechnology Co., Ltd, Building 8, No.12, Lane 855, Jinzheng Road, Jinshan Industrial Park, Shanghai, China

**Keywords:** Bispecific antibodies, Non-protein A purification, Byproducts, Homodimer

## Abstract

Bispecific antibodies hold significant potential as next-generation biotherapeutics owing to their ability to simultaneously bind to two targets. However, the development of bispecific antibodies as biotherapeutics has been hindered by the high levels of byproducts produced, including both high molecular weight and low molecular weight variants. In addition, the inevitable expression of homodimers in host cells presents further obstacles to the commercial development of bispecific antibodies as therapeutics. These byproducts, which share similar physicochemical properties with the target, pose several challenges for downstream purification processes. In this study, we present a non-protein A purification platform that employ a one-step polishing chromatography to purify bispecific antibodies. Mixed-mode Capto adhere resin was used to capture the target protein at pH 7.90 ± 0.10, followed by anion exchange chromatography as a polishing step. Overall, the results of this two-step chromatography purification method demonstrated at final product purity of 98% as assessed by size-exclusion high-performance liquid chromatography (SEC-HPLC) and 98% by reversed-phase-high-performance liquid chromatography (RP-HPLC), with residual host cell proteins controlled at 10 ppm and an excellent recovery rate of approximately 60%. This study presents a non-protein A capture platform, offering a simplified, streamlined, and competitive alternative to conventional affinity chromatography.

**Graphical Abstract:**

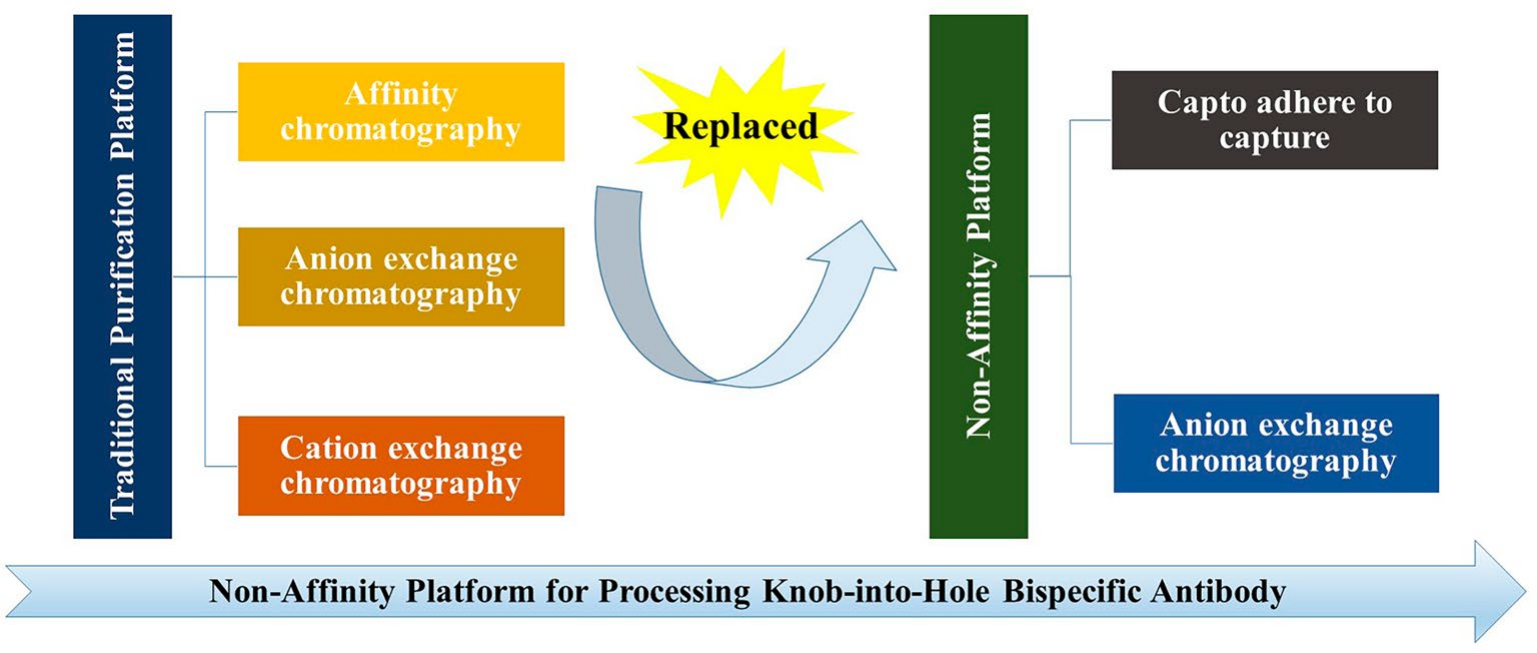

**Supplementary Information:**

The online version contains supplementary material available at 10.1186/s40643-024-00827-8.

## Introduction

Bispecific antibodies (bsAbs) are antibodies capable of simultaneously binding to two different epitopes or targets, thereby enhancing the selectivity and functional affinity of antibodies and improving the efficacy of drugs (Labrijn et al. 2019; Li 2019; Li et al. 2020). As an emerging class of therapeutic antibodies, bsAbs represent a rapidly advancing field in pharmaceutical research and development. Based on their structural formats, bsAbs can be categorized into two main types: IgG-like molecules, which include an Fc region, and non-IgG-like molecules, which lack the Fc region (Guo et al. 2020). The IgG-like molecules can be further categorized into two subgroups based on their structural symmetry: symmetric and asymmetric. The majority of bispecific IgG molecules are asymmetric, whereas IgG fusion proteins often exhibit symmetric molecular compositions (Brinkmann and Kontermann 2017; Li et al. 2020). Typically, asymmetry involves two different heavy chains and two different light chains (Chen and Zhang 2021). Owing to the random assembly of these chains, misassembled species may account for approximately 90% of the total mass (Guo et al. 2020; Li et al. 2020). Homodimers, which possess physicochemical properties similar to those of intact antibodies, present a challenge for separation from the target product. Moreover, bsAbs are more prone to aggregation than monoclonal antibodies (mAbs), complicating the downstream purification process aimed at removing these aggregates.

To minimize the formation of byproducts, various molecular design strategies have been developed to promote correct chain pairing, including the knob-into-hole, CrossMab, and DuetMab methods. Among these, the knob-into-hole approach is the most well-established and widely applied (Chen et al. 2022b). In this approach, a "knob" is created in one CH3 domain by replacing a small amino acid with a larger one, while a "hole" is created in the other CH3 domain by replacing a large residue with a smaller one (Chen et al. 2019; Li 2021). While knob-into-hole bsAbs effectively reduce the formation of homodimers, hole–hole dimers may still form at low levels, and knob–knob homodimers occur even more rarely due to the inherent steric hindrance posed by the knobs (Chen et al. 2022b). Therefore, challenges remain in downstream processing. The development of chromatographic purification processes in biopharmaceutical manufacturing is highly challenging and complex, particularly during early stages. To enhance the efficiency of downstream antibody development, high-throughput process development (HTPD) is increasingly employed. Currently, standard HTPD tools include microwell plates (e.g., PreDictor 96-well plates), which offer speed and minimal sample consumption, as well as larger-volume RoboColumn® units, which enable more precise investigations of column-based mass transfer and operational (pressure-flow) properties(Coffman et al. 2008).

At present, the most used purification strategy involves affinity chromatography, followed by two polishing steps that primarily remove aggregates and hole–hole dimers. However, protein A chromatography has certain limitations, such as harsh elution conditions, high cost, and ligand leakage (Maria et al. 2015; Kateja et al. 2018). Recently, ion exchange chromatography (IEX) and hydrophobic interaction chromatography (HIC) have emerged as promising candidates for innovative antibody purification, offering potential replacements for affinity chromatography in protein capture (Li et al. 2022; Liang et al. 2023). Nikhil Kateja et al. employed cation exchange chromatography (CEX) for the capture of mAbs, achieving more than a tenfold reduction in host cell proteins (HCPs) and DNA, as well as over a 1.5-fold reduction in high molecular weight species (HMWs) during this step (Kateja et al. 2018). In this study, we present a non-protein A purification platform for knob-into-hole (KIH) bsAbs. Initially, HTPD was used to quickly identify Capto adhere as an alternative to protein A resin. Following this, suitable anionic resins and loading conditions were screened. A two-step chromatography platform was subsequently applied to purify the bsAb with a knob-into-hole design. Typically, the charges and hydrophobicity of homodimers differ from those of heterodimers, as homodimers are less well-folded (Chen et al. 2019). Capto adhere was utilized to capture the target protein while removing byproducts, including aggregates, hole–hole dimers, and HCPs. To further improve product quality, we employed anion exchange chromatography (AEX) with a weak binding mode as an additional polishing step. High-throughput screening was conducted to select the appropriate resin and loading conditions. By optimizing pH and conductivity and utilizing AEX in weak binding mode, AEX was able to effectively remove HCP and enhance product purity (Kelley et al. 2008). The final results demonstrated an improvement in purity to 98%, as measured by size exclusion high-performance liquid chromatography (SEC-HPLC) and reversed-phase high-performance liquid chromatography (RP-HPLC), with residual HCPs reduced to < 10 ppm. The procedure described in this study can serve as a reference for removing hole–hole dimers from bsAbs.

## Material and methods

### Antibody and their characteristics

**Fig. 1:**
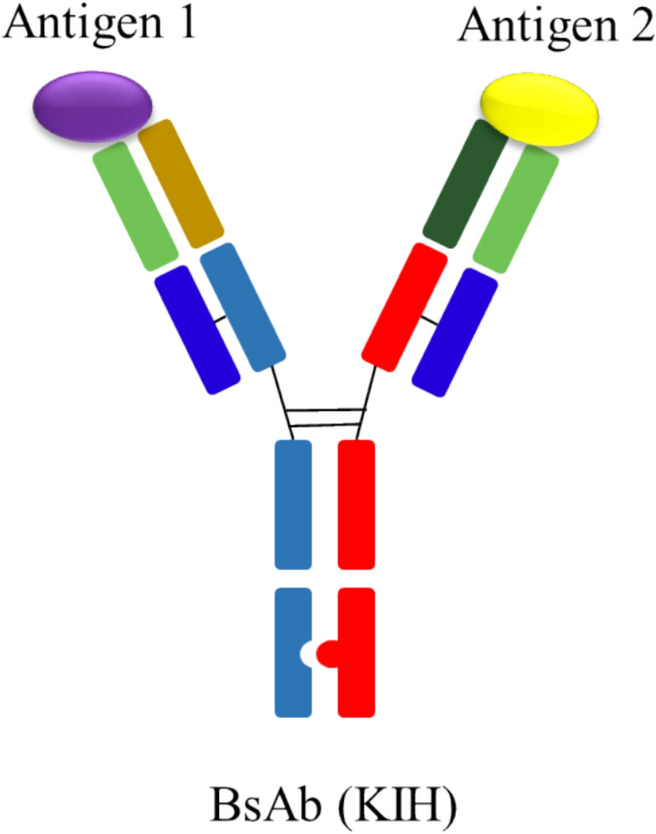
The structure of KIH molecule and the interaction between the KIH molecule and two different antigens

The bsAb was expressed in Chinese hamster ovary (CHO) cells using standard cell culture techniques in a stirred bioreactor. The cultivation was performed in 2-L Biostat® bioreactors (Sartorius Stedim Biotech, Göttingen, Germany) in batch mode for 14 days. Cell clarification was achieved through a two-step centrifugation process, followed by filtration using a Sartoclear BT 1000 filter with a pore size of 0.22 µm (Sartorius Stedim Biotech). The bsAb contain two different high chains and two same light chains, and involves mutating a small amino acid in the CH3 region of one HC of the bispecific antibody into a larger amino acid, thereby creating a prominent type structure. Simultaneously, a large amino acid in the CH3 region of the other heavy chain is mutated into a smaller amino acid, resulting in a depressed "holes" type structure (Chen et al. 2022b). This design leverages the steric hindrance effect of the "KIH" structure to facilitate the correct assembly of the two different heavy chains (Fig. 1). The antibody characteristics were shown in Table 1.

**Table 1:** Antibody characteristics of KIH bsAbs

Molecule type	Molecular weight (kDa)	Isoelectric point		
		HK	HH	KK
BsAb (KIH)	145	7.94	7.45	8.20

*HK* Intact antibody, *HH* hole–hole dimer, *KK* knob–knob dimer

The pI is a theoretical value and is predicted by ExPASy

### ÄKTA purification system

The ÄKTA Pure 150 System equipped with Unicorn software version 7.3 (Cytiva, Uppsala, Sweden) was used for preparative column chromatography.

### Resin screening

To efficiently screen suitable resins for protein capture, we selected four resins for comparison under different loading conditions. The resins included Diamond Q (BESTCHROM, cat. AI0111, China), POROS 50HQ (Thermo, cat. 1-2559-11, USA), NanoGel 50Q (NanoMicro, cat. 04064–050200, China), and Capto adhere (Cytiva, cat. 10302163, USA). The pH levels were adjusted to 7.6, 7.7, 8.0, and 8.2, while the conductivity was set to 3.0, 5.0, and 8.0 mS/cm. A total of 230 μL of resin (solid–liquid ratio 1:1) was added to 96-well filter plates. All resins were equilibrated three times with the respective equilibration buffer (50 mM Tris-HAc). The sample (concentration: 3.18 g/L) was then added to the corresponding AcroPrep Advance 96-well filter plate (Cytiva) and incubated under shaking conditions (2 h, room temperature, 1100 rpm). The flow through was collected by centrifugation at 300 × *g* for 1 min. Subsequently, the flow through was analyzed using SEC-HPLC and RP-UPLC. Protein concentration was measured using a NanoDrop spectrophotometer (Implen NanoPhotometer N60, Germany), and the adsorption capacity was calculated using the following equation:

$${\text{Q}}\text{*}=\frac{\left({C}_{0}- {C}^{*}\right) \, \text{V }}{{V}_{R}}$$

Here, Q* denotes the protein adsorption capacity per unit volume of resin (mg/mL); *C*_*0*_ represents the initial protein concentration (mg/mL); C* denotes the protein concentration at equilibrium (mg/mL); *V* represents the volume of protein solution (mL); and *V*_*R*_ indicates the volume of the resin (mL).

### Parameters for mix mode chromatography (MMC)

MMC chromatography was employed for capture using Capto adhere (Cytiva, Cat. No. 10302163, USA) in the binding-elution mode, utilizing an anion mixed-mode resin. Prior to the experiment, the pH of the loading sample was adjusted to 7.9 ± 0.1, and the conductivity was lower to below 5.0 mS/cm. The conductivity of the cell culture fluid (CCF) was approximately 10.0 mS/cm when diluted with pure water. Doubling the dilution had no significant impact on scale-up commercial production.

The sample was harvested from the CCF containing bsAb. The resin had loading capacity of 40 g/L. The column was equilibrated with 50 mM Tris-HAc at pH 7.9 for 3 column volumes (CVs). After loading the sample, we washed the column with 50 mM Tris-HAc at pH 7.9. The product was then eluted using a gradient over 20 CVs, employing 50 mM Tris-HAc at pH 7.9 and 50 mM NaAc-HAc at pH 5.5. The column information was shown in Table 2.

**Table 2:** Volume and flow rate for MMC

Resin	Inner Diameter (cm)	Bed Height (cm)	Column Volume (mL)	Linear Flow Rate (cm/h)
Capto adhere	0.66	20.5	7.0	245.5

### Dynamic binding capacity measurement for Capto adhere

Dynamic Binding Capacity (DBC) of the chromatographic device at 5% break-through (DBC_5%_) was assessed on the ÄKTA Pure 150 System. For Capto adhere, product load residence time of 5 min was studied, the titer of initial CCF was 3.18 g/L. To ensure product breakthrough, Capto adhere was loaded to 80 g/L. Before the experiment, the pH of the loading sample was adjusted to 7.9 ± 0.1, and the conductivity was reduced to below 5.0 mS/cm. The detailed information was shown in Table 3.

**Table 3:** Volume and flowrate for determination of DBC_5%_

Resin	Inner Diameter (cm)	Bed Height (cm)	Column Volume (mL)	Linear Flow Rate (cm/h)
Capto adhere	0.66	20.5	7.0	245.5

### Parameters of AEX

AEX was performed in weak binding mode using NanoGel 50Q resin (NanoMicro), with the sample derived from MMC. Prior to sample loading, the column was pre-equilibrated with 50 mM Tris-HAc and 150 mM NaCl at pH 7.4, followed by further equilibration with 50 mM Tris-HAc at pH 7.8 for at least 3 CV, respectively. The pH of the loading sample was adjusted to 7.8 ± 0.1 using 1 M Tris, and the conductivity was reduced to below 3.0 mS/cm. The loading capacity ranged from 50 to 120 g/L of resin. After loading, the column was washed with 50 mM Tris-HAc at pH 7.8. The pH of the collected eluates was measured using an external pH probe (Mettler Toledo). The column information was shown in Table 4.

**Table 4:** Column information for AEX

Resin	Inner Diameter (cm)	Bed Height (cm)	Column Volume (mL)	Linear Flow Rate (cm/h)
NanoGel 50Q	0.66	20.5	7.0	245.5

### Large-scale validation for MMC and AEX

Capto adhere was used to performed the capture step. Before the experiment, the pH of the loading sample was adjusted to 7.90 ± 0.10, and the conductivity was reduced to below 5.0 mS/cm. The sample was harvested from the CCF containing bsAb. The resin's loading capacity was 40 g/L. The column was equilibrated with 50 mM Tris-HAc at pH 7.9 for 3 column volumes. After loading the sample, we washed the column with 50 mM Tris-HAc at pH 7.9. The product was then eluted using a gradient elution (0–100% B) over 20 CVs with 50 mM Tris-HAc solution at pH 7.9 (A buffer) and 50 mM NaAc-HAc solution at pH 5.5 (B buffer). The column information was shown in Table 4.

NanoGel 50Q was used to perform AEX. Before loading, the column was pre-equilibrated with 50 mM Tris-HAc and 150 mM NaCl at pH 7.4, followed by equilibration with 50 mM Tris-HAc at pH 7.8 for at least 3 CV, respectively. The pH of the loading sample was adjusted to 7.8 ± 0.1 using 1 M Tris, and the conductivity was reduced to less than 3.0 mS/cm. After loading, the column was washed with 50 mM Tris-HAc at pH 7.8. The pH of the collected eluates was measured using an external pH probe (Mettler Toledo). The column information was shown in Table 5.

**Table 5:** Column information for MMC and AEX

Resin	Inner Diameter (cm)	Bed Height (cm)	Column Volume (mL)	Linear Flow Rate (cm/h)
Capto adhere	10.0	16.0	1256.0	192.0
NanoGel 50Q	5.0	25.5	500.4	305.9

### SEC-HPLC analysis

A SEC-HPLC analysis was conducted using an AdvanceBio SEC 300A, 2.7 μm, 7.8 × 300 mm column (Agilent Technologies, Cat. No. 0006663018–18). The mobile phase consisted of 100 mM sodium phosphate, 250 mM NaCl, and 5% isopropanol at pH 6.8. Each bsAb sample was injected at a protein amount of 50 μg. Isocratic elution was performed at a flow rate of 0.8 mL/min and a temperature of 30 °C, with protein elution monitored by ultraviolet (UV) absorption at 280 nm.

### RP-HPLC analysis

The molecular weight and isoelectric point (pI) of the hole–hole dimer and the bsAb are remarkably similar, making it challenging for SEC-HPLC, ion exchange-high-performance liquid chromatography (IEX-HPLC), and imaged capillary isoelectric focusing (iCIEF) to effectively distinguish between them. Therefore, RP-HPLC was introduced to evaluate the removal of hole–hole dimers.

All samples were analyzed using an ACQUITY™ Premier Protein BEH C4 column (1.7 μm, 2.1 × 150 mm; Waters, Cat. No. 0158331660220). The mobile phase consisted of 0.1% trifluoroacetic acid (TFA) in water and 0.1% TFA in acetonitrile (ACN). The injection volume of bsAb was 10 μg per sample. Elution was performed isocratically at a flow rate of 0.4 mL/min and a temperature of 70 °C. Protein elution was monitored by UV absorption at 280 nm.

### Residual HCP measurement

HCP was detected using a sandwich enzyme-linked immunosorbent assay (ELISA) with the Cygnus CHO Host Cell Proteins 3rd Generation Kit. A 50 µL sample and 150 µL of α-CHO: Horseradish Peroxidase (HRP) were added to the coated 96-well microtiter strips and incubated at 400–500 rpm for 2 h. The strips were then washed three times with 300 µL of 1 × washing buffer per wash. Next, 100 µL of 3,3′,5,5′-tetramethylbenzidine (TMB) color-developing solution was added, and the plates were kept protected from light for 2 h. Finally, 50 µL of stop solution was added, and the absorbance was measured using a microplate reader at an optical density (OD) of 450–650 nm.

## Results and discussion

### HTPD preliminary evaluation of the performance of mixed-mode resin and ionic resins for bsAb molecules

The proposed scheme employs IEX or HIC as the capture step rather than protein A chromatography. In this study, it was necessary to determine the appropriate IEX resin, as well as the optimal loading conditions (Kateja et al. 2018; Li et al. 2022). In general, the pH of the sample in AEX and anionic hydrophobic mixed-mode with binding mode should be higher than the pI of the sample. This ensures that there is no need to adjust the pH to lower condition, which could lead to protein instability due to acidic conditions. Considering these factors, AEX and anionic hydrophobic mixed-mode resins were selected for protein capture.

**Fig. 2:**
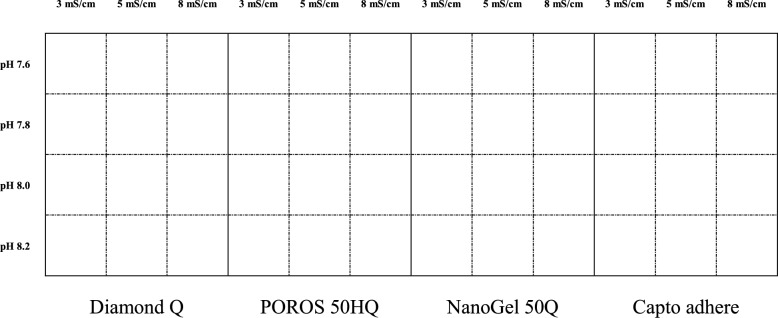
Schematic diagram illustrating the comparison of resins, loading pH, and conductivity with the HTPD platform for the purification of KIH bsAb protein

HTPD can be used for the initial screening of process conditions, effectively shortening development timelines. To rapidly determine capture resin and loading conditions for KIH bsAb, we used HTPD for preliminary exploration. In this study, we employed a 96-well filter plate-based HTPD method to conduct an initial evaluation of purification performance. As shown in Fig. 2, three parameters were investigated: resin type, pH, and conductivity. Three AEX resins were selected—Diamond Q, POROS 50HQ, and NanoGel 50Q, along with a mixed-mode resin, Capto adhere. The pIs of the intact antibody (HK), hole–hole dimer (HH), and knob–knob dimer (KK) were determined to be 7.94, 7.45, and 8.20, respectively. Based on the differences in pIs and the characteristics of the AEX resins, we selected loading pH values close to the pIs of the proteins to facilitate the separation of the homodimer and HK. The selected pH conditions were 7.6, 7.8, 8.0, and 8.2. In addition, conductivity is a key parameter for AEX resins, as lower conductivity generally promotes tighter sample binding. Conductivities of 3.0, 5.0, and 8.0 mS/cm were tested.

Initially, under the pre-set conditions, only Capto adhere was able to capture the KIH molecule, while samples in the other three AEX resins passed through without binding. This outcome can be attributed to the insufficient binding strength of the product to the resin, which relies solely on ionic interactions at a pH near the pI, necessitating higher pH conditions. In contrast, Capto adhere offers not only ionic interactions but also hydrophobic and hydrogen bond interactions, allowing the product to be captured through multiple interactions at a pH close to the pI (Chen et al. 2022a). Based on these results, Capto adhere was selected for capturing the KIH molecule in this study.

Capto adhere is a mixed-mode resin with strong ion exchange properties that enables hydrophobic interactions, hydrogen bonding, and electrostatic interactions (O’Connor et al. 2017). Capto adhere not only effectively removes impurities and viruses in flow-through mode but also improves sample purity and efficiently eliminates HCPs and viruses in bind-elute mode. However, the increased complexity of multimodal resins necessitates more process optimization to fully leverage the remarkable potential of this technology. HTPD offers an efficient means to facilitate this optimization, with advantages including reduced solvent consumption, high productivity, and significant time savings (Coffman et al. 2008).

As shown in Table 6, within the pH range of 7.6–8.0, the Q* value decreased as conductivity increased. However, the pH change itself had minimal effect on the Q* value, indicating that Capto adhere primarily captured the protein through ionic interactions at this stage. A significant decrease in the Q* value was observed when conductivity reached 8.0 mS/cm within this range (Supplementary data 1). Conversely, when the pH to 8.2, the Q* value increased with a rise in conductivity at 8.0 mS/cm, indicating that Capto adhere primarily captured the sample through hydrophobic interactions. In this study, the removal of by-products was primarily achieved by exploiting the pI difference between the homodimer and the product. Based on these results, the loading conditions for Capto adhere could be set to a pH of 7.6–8.0 and a conductivity of ≤ 5.0 mS/cm.

**Table 6:** Q* values for bsAb in the Capto adhere resin

pH	Conductivity (mS/cm)	Q* (mg/mL)
7.6	3.0	26.65
7.6	5.0	25.32
7.6	8.0	21.97
7.8	3.0	29.30
7.8	5.0	28.91
7.8	8.0	22.26
8.0	3.0	26.81
8.0	5.0	25.94
8.0	8.0	24.13
8.2	3.0	15.31
8.2	5.0	19.83
8.2	8.0	25.33

Q* values: protein adsorption capacity per unit volume of resin (mg/mL)

Mixed-mode resins are commonly used in capture chromatography across various applications and have so far exhibited excellent performance. Our data suggest that bsAbs can be effectively captured through the bind-elute mode using Capto adhere.

**Fig. 3:**
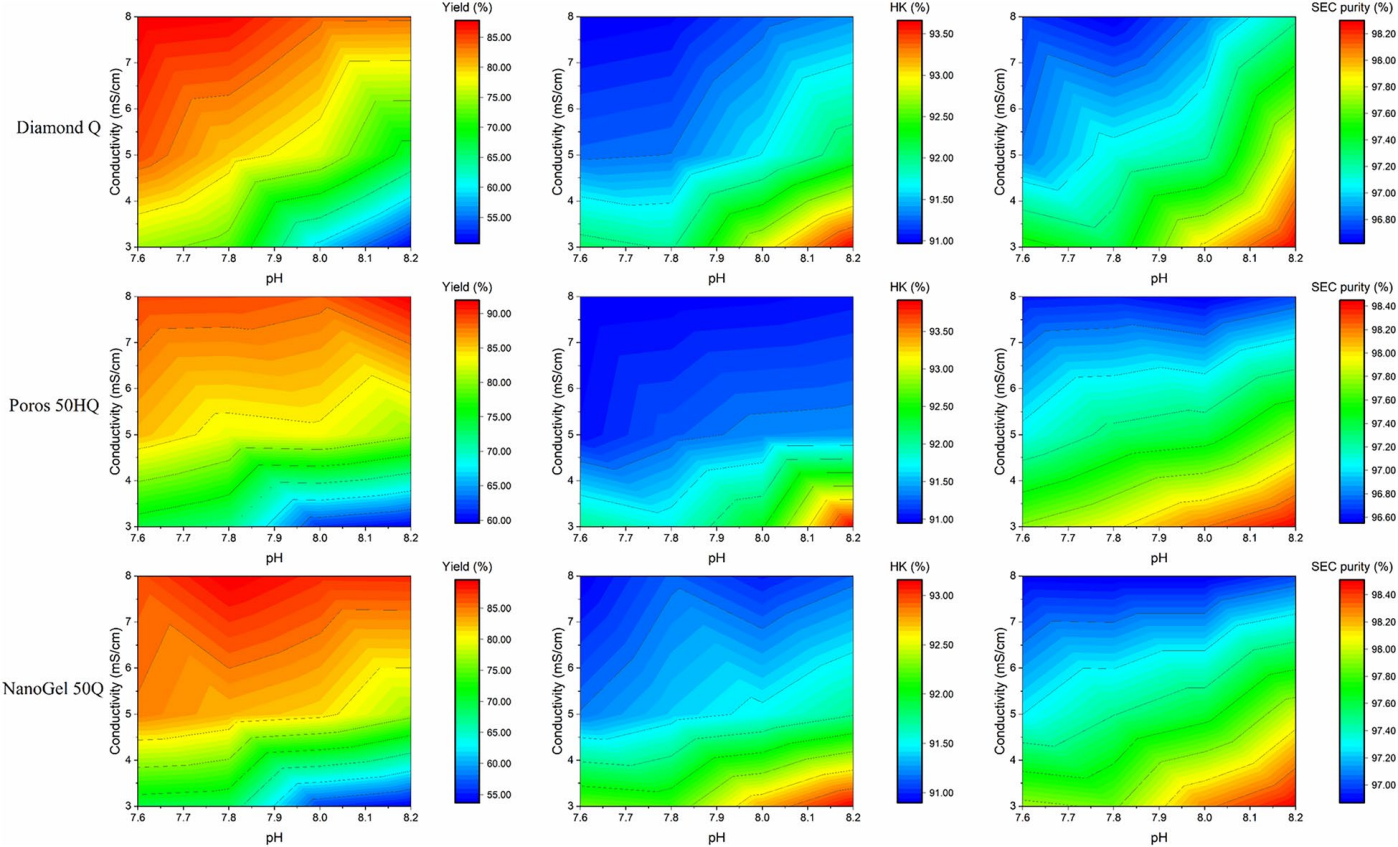
Resin comparison results under different conditions. Compared with the KIH bsAb’s yield and purity of SEC-HPLC and RP-HPLC for three AEX resins

Although the three AEX resins were unable to capture KIH antibody molecules at pH 7.6 –8.2 and a conductivity of ≤ 8.0 mS/cm, the flow-through samples were analyzed using SEC-HPLC and RP-HPLC, and the yields were compared. The experimental results were shown in Fig. 3.

Similar trends in yield and purity were observed across the different resins under the investigated conditions. The initial purity of SEC-HPLC and RP-HPLC was 95.21% and 82.02%. Impurity removal was effectively achieved in flow-through mode using all three resins under these conditions. An increase in pH, combined with decreased conductivity enhanced the purity of the target molecule but compromised the yield. Among the resins, Diamond Q exhibited relatively low monomer purity (approximately 96%). In contrast, NanoGel 50Q demonstrated superior purification performance, with monomer purity at around 98% and target bsAb purity at approximately 92%. This result is related to the properties of the resins, the particle size of Diamond Q is larger than that of NanoGel 50Q and POROS 50HQ; thus, Dimond Q's resolution is lower than that of NanoGel 50Q and POROS 50HQ, resulting in the lower purity for Diamond Q. In terms of the support matrix, NanoGel 50Q is monodisperse, with better matrix separation due to its monodisperse structure (Supplementary data 2). As a result, the product purity is better for NanoGel 50Q. However, higher conductivity was found to reduce the purity of the target. Considering both yield and purity, the conditions of pH 7.8 and conductivity of 3.0 mS/cm were found to be optimal for NanoGel 50Q, resulting in a target bsAb purity of 97.9%. These HTPD results suggested that NanoGel 50Q has potential as an AEX resin for bsAb purification.

Our results showed that AEX effectively removed homodimers, aggregates, and fragments under weak binding conditions. Adjustments in pH and conductivity produced opposing trends in purity and yield rates. Therefore, we selected a pH of 7.8 ± 0.2 and a conductivity of < 3.0 mS/cm as the loading conditions to balance yield and product quality. Weak partitioning in AEX proved to be a superior polishing strategy for achieving high heterodimer purity.

HTPD rapidly identified that Capto adhere can capture KIH bsAbs under loading conditions of pH 7.8 ± 0.2 and a conductivity of ≤ 5.0 mS/cm. This discovery paves the way for further optimization of loading conditions, potentially reducing process development time. In addition, although the three AEX resins were unable to capture the protein, purity could still be improved through a weak binding mode within a pH range of 7.6–8.2. By screening resins and loading conditions using HTPD, the appropriate anion resin and optimal loading parameters were identified. Consequently, HTPD demonstrates its effectiveness as a tool for process development, especially for complex molecules.

### Loading condition optimization and DBC determination

**Fig. 4:**
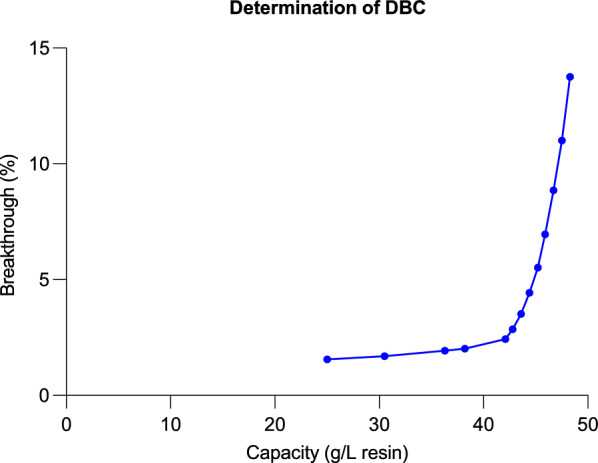
DBC of Capto adhere for KIH bsAb. The abscissa represents the loading capacity, and the ordinate represents the ratio of flow-through sample titer vs. initial titer. Binding capacity at 5% breakthrough was calculated to set the DBC

The Q* values of HTPD represent the mean protein adsorption capacity per unit volume of resin (mg/mL). The resin loading capacities determined by HTPD were based on static conditions, which may not precisely reflect the results under dynamic loading conditions. To further evaluate the DBC of Capto adhere at a pH of 7.8 and a conductivity of 5.0 mS/cm, a breakthrough experiment was conducted to determine the loading capacity of Capto adhere. The volume at which 5% of the load mAU was achieved was defined as the 5% breakthrough volume. The estimated protein loading capacity was 45 mg of protein per mL of resin (Fig. 4).

Based on the HTPD results, a loading condition of pH 7.8 ± 0.2 and conductivity of ≤ 5.0 mS/cm is recommended due to the static loads. However, it is necessary to determine the optional loading condition and acceptable loading range using a chromatography column. The HTPD results indicated that loading pH values were set at 7.6, 7.8, and 8.0, with conductivity maintained at 5.0 mS/cm. Product purity by SEC-HPLC and RP-HPLC, and HCP removal were evaluated, and the results were summarized in Table 7. Based on the results, a pH of 7.9 ± 0.1 and conductivity of ≤ 5.0 mS/cm are suggested as the optimal loading conditions.

**Table 7:** The yield, SEC-HPLC and RP-HPLC, HCP content of different loading pH for Capto adhere

Loading pH	Yield (%)	SEC-HPLC (%)			RP-HPLC (%)		HCP (ppm)
		HMW	Main	LMW	HH	HK	
7.6	65.23	2.63	96.93	0.44	2.87	97.13	2157.98
7.8	73.40	2.50	97.33	0.17	2.39	97.61	2315.67
8.0	75.57	0.63	97.93	1.44	2.57	97.43	2743.24

Based on the aforementioned results, the optimal loading conditions identified by HTPD provide a screening range for column chromatography loading conditions, effectively reducing the time required to identify suitable conditions. In addition, although the Q* values of HTPD and DBC differed. Q* value is a loading capacity that was tested on static conditions, it only can demonstrate Capture adhere can capture the target protein, and the loading conditions for capture are clearly defined but may not precisely correspond to the results of dynamic loading capacities.

### Determination of loading capacity for AEX

To further reduce HCPs and improve product purity in SEC-HPLC and RP-HPLC, additional polishing steps are necessary. In downstream processes, an AEX flow-through not only removes HCPs and host cell DNA (HCD) but also serves as an effective viral clearance step. Therefore, a one-step AEX flow-through is recommended following capture chromatography (Masuda et al. 2020).

The flow-through mode of AEX is commonly used as a polishing step for antibodies. It is generally considered more effective at removing HCPs or HCD than at eliminating product-related impurities, such as hole–hole dimers and aggregates. This study demonstrated that a single-step AEX process can effectively remove both aggregates and hole–hole dimers, suggesting that a weak binding mode may offer a better polishing strategy for achieving high heterodimer purity. Therefore, in this study, the determination of AEX loading capacity differed from the general flow-through mode and was evaluated not only based on the removal capacity for HCPs but also according to the removal efficiency and yield of aggregates and hole–hole dimers.

Based on the experimental results obtained from HTPD, the appropriate AEX resin and optimal loading conditions were identified. To confirm the loading of KIH molecules in NanoGel 50Q resin, we evaluated SEC-HPLC and RP-HPLC purity and yield at four different loading capacities (30, 50, 100, and 150 g/L), with a retention time of 5 min. The sample loading conditions were set at pH 7.8 and a conductivity of 3.0 mS/cm. The experimental results were shown in Table 8. The experimental results indicated that the product yield was 75.42% at a loading capacity of 30 g/L, with SEC-HPLC and RP-HPLC purities of 99.51% and 99.7%, respectively, and an HCP content of 5.32 ppm, although the quality data is acceptable, the yield is too low. Moreover, the lower limit for comprehensive quality data and yield was set at a loading capacity of 50 g/L. At 150 g/L, the SEC-HPLC purity was 99.01%, and aggregate removal was decreased compared with the results at 100 g/L. Consequently, the upper loading capacity was set at 150 g/L. To balance the yield and quality data, the final capacity range for the anion weak binding mode was determined to be 50–150 g/L.

**Table 8:** Results of AEX loading capacity determination

Loading Capacity (g/L)	Yield (%)	SEC-HPLC (%)			RP-HPLC (%)		HCP (ppm)
		HMW	Main	LMW	HH	HK	
30	75.42	0.42	99.51	0.07	0.3	99.7	5.2
50	87.64	0.51	99.39	0.10	0.3	99.7	3.3
100	89.86	0.75	99.18	0.07	0.3	99.7	6.6
150	90.10	0.91	99.01	0.08	0.3	99.7	8.2

Compared with the flow-through mode of AEX, the weak binding mode offers enhanced removal of aggregates and hole–hole dimers, along with the removal of HCPs and HCD. If the loading capacity is too low, the resin can capture a sufficient amount of impurities but still bind to some of the product, potentially reducing the product yield. However, if the loading capacity is too high, impurities may pass through, resulting in a higher product yield but lower product quality. Therefore, it is necessary to balance the mass and yield when determining the optimal load in the anion weak binding mode.

### Large-scale validation

The previous study comprised a small-scale experiment on a 7 mL column. A 10-L scale validation was subsequently performed to demonstrate the scalability of these experiments.

Initially, Capto adhere was used for capture at pH 7.90 and a conductivity of 5.0 mS/cm, with a resin loading capacity of 30 g/L. A 1.2-L chromatography column was employed, and the results of the Capto adhere experiment are shown in the table. The yield was 73.44%, the purity determined by SEC-HPLC was 97.50%, the purity by RP-HPLC was 98.48%, and the HCP content was 1878 ppm. These results are comparable with those obtained in previous experiments conducted on small-scale columns.

Subsequently, we eluted samples from Capto adhere in weak binding mode AEX using a 0.5 L column at pH 7.8 and a conductivity of 3.0 mS/cm. As shown in Table 9, the yield after AEX was 89.09%, the SEC-HPLC purity was 98.80%, the RP-HPLC purity was 98.76%, and the HCP content was 3.4 ppm. These scale-up results are consistent with those obtained from small-scale experiments.

**Table 9:** The yield, SEC-HPLC, RP-HPLC, and HCP content of CCF, Capto adhere eluate and NanoGel 50Q FT for large-scale validation

Sample name	Yield (%)	SEC-HPLC (%)			RP-HPLC (%)		HCP (ppm)
		HMW	Main	LMW	HH	HK	
CCF	N/A	3.56	95.21	1.23	17.98	82.02	103,698.91
Capto adhere eluate	73.44	2.32	97.50	0.18	1.39	98.61	2315.67
NanoGel 50Q FT	89.09	0.41	98.80	ND	1.43	98.57	7.49

*CCF* cell culture fluid. *ND* not detected

Based on these results, the non-protein A two-step purification platform can be scaled up to a 10-L process. After establishing a process lock at this scale, the method demonstrates potential for further scaling to larger production volumes, such as 200 L, 500 L, or beyond.

Antibody purification platforms typically require multiple steps to effectively remove viruses. These steps may include affinity chromatography, low pH viral inactivation, AEX, and virus filtration. In this study, the product quality results met the required standards. According to literature suggests that Capto adhere is effective in clearing viral clearance, offering a viable alternative to affinity chromatography (Brown et al. 2018). Therefore, no additional polishing steps are necessary.

## Conclusion

In the purification of bsAbs, Capto adhere can be effectively utilized to capture the protein from CCF. This approach not only removes byproducts and HCP but also serves as a step for viral clearance. For mixed-mode resins, the application of HTPD can efficiently assist in optimizing the loading conditions. According to the results of HTPD, when the pH is 7.6–8.0 and the conductivity ≤ 5.0 mS/cm, the Q* of the antibody will decrease, and combined with the column chromatography experiment, it is found that when the pH is 7.6, the product yield decreases, so the loading conditions of Capto adhere are recommended to be pH 7.9 ± 0.1 and conductivity ≤ 5.0 mS/cm. During the polishing step, the use of AEX in weak binding mode allows for the flow-through of intact bsAbs, a pH of 7.8 ± 0.2 and a conductivity of < 3.0 mS/cm as the loading conditions to balance yield and product quality. Following the two-step chromatography process, we successfully demonstrated the efficient capture of target molecules and excellent removal of byproducts and impurities. This method produces a final product with high purity, as measured by SEC-HPLC (> 98%) and RP-HPLC (> 98%), with low impurity levels (HCP < 10 ppm) and a robust overall recovery rate of approximately 60%. This represents a notable improvement from the monomer purity of approximately 60% observed in the CCF. These results underscore the feasibility and effectiveness of the non-affinity capture platform in challenging bsAb downstream processing, particularly its ability to remove bsAb-specific byproducts, such as hole–hole homodimer mispairing products. The non-affinity chromatography and AEX weak binding mode polishing methods for homodimers and aggregates may serve as a reference for the removal strategies of other contaminants in bsAb purification.

## Supplementary Information

Supplementary Material 1.

## Data Availability

All data generated or analyzed during this study are included in this article.

## Abbreviations

AEX: Anion exchange chromatography
bsAb: Bispecific antibody
CCF: Cell culture fluid
HMW: High molecular weight
LMW: Low molecular weight
HCP: Host cell protein
HCD: Host cell DNA
HTPD: High-throughput process development
OD: Optical density
KIH: Knob-into-hole
HK: Intact antibody
HH: Hole-hole dimer
KK: Knob-knob dimer
SEC: Size exclusion chromatography
RP: Reversed-phase
IEX: Ion exchange
HPLC: High performance liquid chromatography
CHO: Chinese hamster ovary
CV: Column volume
UV: Ultra-violet
pI: Isoelectric point
iCIEF: Imaged capillary isoelectric focusing
HRP: Horseradish peroxidase
DBC: Dynamic binding capacity
ND: Not detected
TMP: 3, 3′,5,5′-Tetramethylbenzidine
Main: Main peak ratio
MMC: Mix mode chromatography

## References

[CR1] Brinkmann U, Kontermann RE (2017) The making of bispecific antibodies. Mabs 9:182–212. 10.1080/19420862.2016.1268307 28071970 10.1080/19420862.2016.1268307 PMC5297537

[CR2] Brown MR, Burnham MS, Johnson SA, Lute SC, Brorson KA, Roush DJ (2018) Evaluating the effect of in-process material on the binding mechanisms of surrogate viral particles to a multi-modal anion exchange resin. J Biotechnol 267:29–35. 10.1016/j.jbiotec.2017.12.018 29278725 10.1016/j.jbiotec.2017.12.018

[CR3] Chen SW, Zhang W (2021) Current trends and challenges in the downstream purification of bispecific antibodies. Antib Ther 4:73–88. 10.1093/abt/tbab007 34056544 10.1093/abt/tbab007 PMC8155696

[CR4] Chen T, Han J, Guo G, Wang Q, Wang Y, Li Y (2019) Monitoring removal of hole-hole homodimer by analytical hydrophobic interaction chromatography in purifying a bispecific antibody. Protein Expr Purif. 10.1016/j.pep.2019.105457 31344474 10.1016/j.pep.2019.105457

[CR5] Chen SW, Hoi KM, Mahfut FB, Yang Y, Zhang W (2022a) Effective flow-through polishing strategies for knob-into-hole bispecific antibodies. Bioresourc Bioprocess. 10.1186/s40643-022-00590-8 10.1186/s40643-022-00590-8 PMC10992779 38647877

[CR6] Chen SW, Hoi KM, Mahfut FB, Yang Y, Zhang W (2022b) Excellent removal of knob-into-hole bispecific antibody byproducts and impurities in a single-capture chromatography. Bioresourc Bioprocess. 10.1186/s40643-022-00562-y 10.1186/s40643-022-00562-y PMC10992212 38647639

[CR7] Coffman JL, Kramarczyk JF, Kelley BD (2008) High-throughput screening of chromatographic separations: I. method development and column modeling. Biotechnol Bioeng 100:605–618. 10.1002/bit.21904 18496874 10.1002/bit.21904

[CR8] Guo G, Han J, Wang Y, Li Y (2020) A potential downstream platform approach for WuXiBody-based IgG-like bispecific antibodies. Protein Expr Purif. 10.1016/j.pep.2020.105647 32334139 10.1016/j.pep.2020.105647

[CR9] Kateja N, Kumar D, Sethi S, Rathore AS (2018) Non-protein A purification platform for continuous processing of monoclonal antibody therapeutics. J Chromatogr A 1579:60–72. 10.1016/j.chroma.2018.10.031 30430988 10.1016/j.chroma.2018.10.031

[CR10] Kelley B, Tobler SA, Brown P, Coffman JL, Godavarti R, Iskra T, Switzer M, Vunnum S (2008) Weak partitioning chromatography for anion exchange purification of monoclonal antibodies. Biotechnol Bioeng 101:553–566. 10.1002/bit.21923 18727127 10.1002/bit.21923

[CR11] Labrijn AF, Janmaat ML, Reichert JM, Parren PWHI (2019) Bispecific antibodies: a mechanistic review of the pipeline. Nat Rev Drug Discov 18:585–608. 10.1038/s41573-019-0028-1 31175342 10.1038/s41573-019-0028-1

[CR12] Li Y (2019) A brief introduction of IgG-like bispecific antibody purification: Methods for removing product-related impurities. Protein Expr Purif 155:112–119. 10.1016/j.pep.2018.11.011 30513344 10.1016/j.pep.2018.11.011

[CR13] Li Y (2021) IgG-like bispecific antibody platforms with built-in purification-facilitating elements. Protein Exp Purif. 10.1016/j.pep.2021.105955 10.1016/j.pep.2021.105955 34416361

[CR14] Li Y, Wang Y, Shen P, Zhou W (2020) A roadmap for IgG-like bispecific antibody purification. In: Approaches to the Purification, Analysis and Characterization of Antibody-Based Therapeutics, pp 167–179.

[CR15] Li X, Liu W, Li H, Wang X, Zhao Y (2022) Capture and purification of an untagged nanobody by mixed weak cation chromatography and cation exchange chromatography. Protein Expr Purif. 10.1016/j.pep.2021.106030 34920133 10.1016/j.pep.2021.106030

[CR16] Liang X, He Q, Qin G, Li G, Li Q, Tan H, Wang Z, Fan M, Xu D (2023) Effectively removing the homodimer in bispecific antibodies by weak partitioning mode of anion exchange chromatography. J Chromatogr B. 10.1016/j.jchromb.2023.123767 10.1016/j.jchromb.2023.123767 37270861

[CR17] Maria S, Joucla G, Garbay B, Dieryck W, Lomenech A-M, Santarelli X, Cabanne C (2015) Purification process of recombinant monoclonal antibodies with mixed mode chromatography. J Chromatogr A 1393:57–64. 10.1016/j.chroma.2015.03.018 25805720 10.1016/j.chroma.2015.03.018

[CR18] Masuda Y, Ogino Y, Yamaichi K, Takahashi Y, Nonaka K, Wakamatsu K (2020) The prevention of an anomalous chromatographic behavior and the resulting successful removal of viruses from monoclonal antibody with an asymmetric charge distribution by using a membrane adsorber in highly efficient, anion‐exchange chromatography in flow‐through mode. Biotechnol Progr. 10.1002/btpr.2955 10.1002/btpr.2955 31894893

[CR19] O’Connor E, Aspelund M, Bartnik F, Berge M, Coughlin K, Kambarami M, Spencer D, Yan H, Wang W (2017) Monoclonal antibody fragment removal mediated by mixed mode resins. J Chromatogr A 1499:65–77. 10.1016/j.chroma.2017.03.063 28389094 10.1016/j.chroma.2017.03.063

