# Supplementary material for: Non-affinity platform for processing knob-into-hole bispecific antibody
Source: Bioresour Bioprocess. 2024 Dec 18;11(1):110. doi: 10.1186/s40643-024-00827-8 (PMC11655889; doi:10.1186/s40643-024-00827-8)
Supplement: Supplementary file 1 — Supplementary Material 1. [file 40643_2024_827_MOESM1_ESM.pdf]

Bioresources and Bioprocessing

Non-Affinity Platform for Processing Knob-into-Hole Bispecific Antibody

Xiaoyang Wang, Min Li, Mengting Li, Huoyan Hong, Kai Gao, Puya Zhao *

Shanghai AsymBio Biotechnology Co., Ltd; Building 8, No.12, Lane 855, Jinzheng Road, Jinshan Industrial Park, Shanghai CN; wangxiao-yang0557@asymchem.com.cn; limin0708@asymchem.com.cn; limengting0707@asymchem.com.cn; honghuoyan@asymchem.com.cn; gaokai@asymchem.com.cn; zhaopuya0522@asymchem.com.cn
*Correspondence: zhaopuya0522@asymchem.com.cn

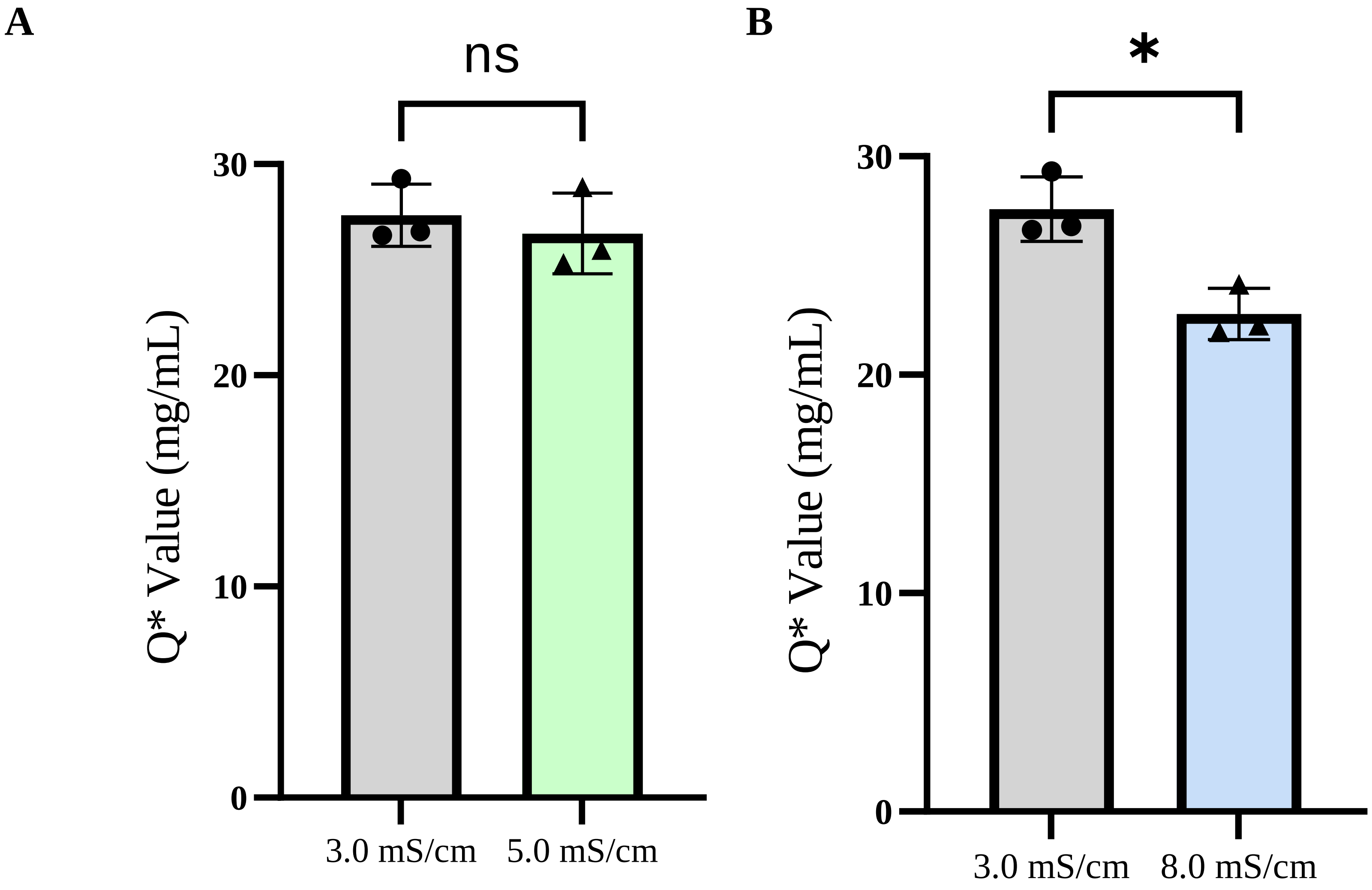

Supplementary Data 1: A : The Q* value comparison results of 3.0 mS/cm and 5.0 mS/cm . B: The Q* value comparison results of 3.0 mS/cm and 8.0 mS/cm. ns: No significant. *: p-value < 0.05, significant.

Resin	Average particle size (μm)	Ligand	Support matrix
NanoGel 50Q	50	Quaternary ammonium salt	Monodisperse polystyrene/divinylbenzene
Diamond Q	90	Quaternary ammonium salt	Agarose
POROS 50HQ	50	Quaternary ammonium salt	Cross-linked poly(styrene-divinylbenzene)

Supplementary Data 2: The properties of NanoGel 50Q, Diamond Q and POROS 50HQ.

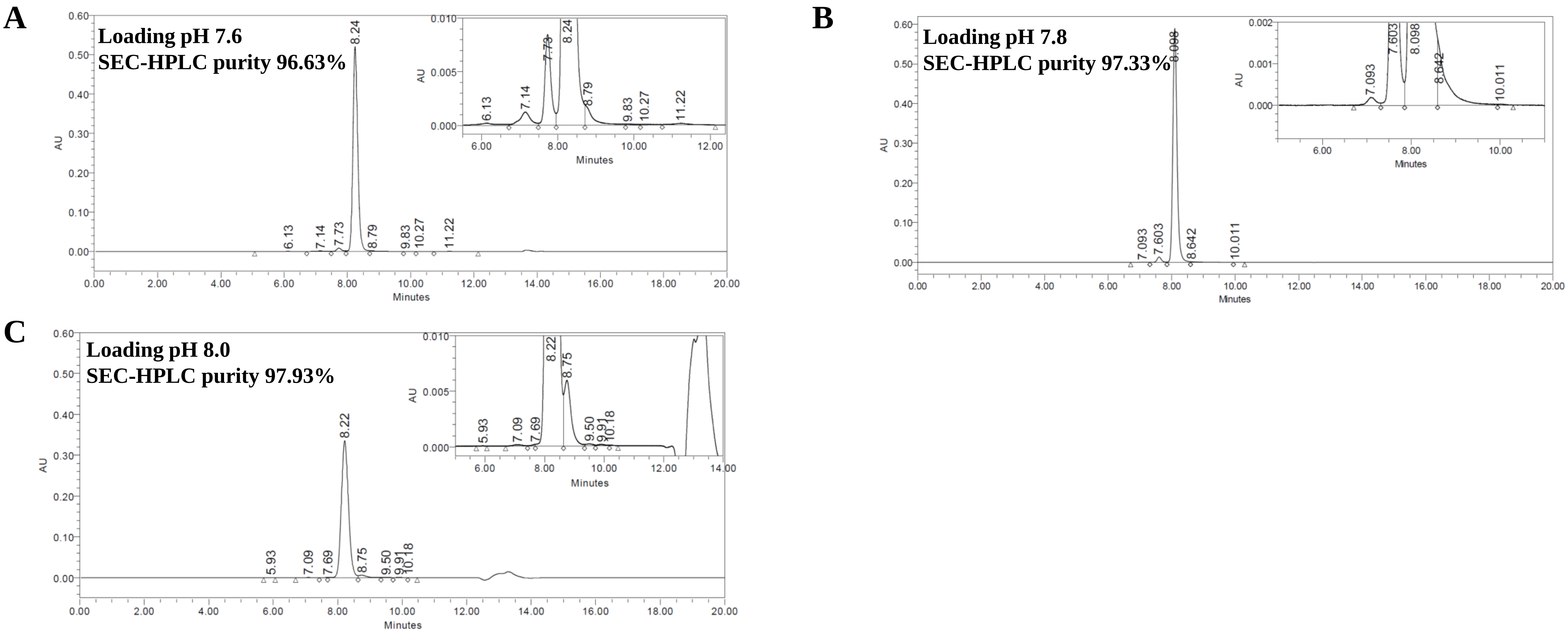

Supplementary Data 3: A : The SEC-HPLC purity of Capto adhere loading condition optimization with pH 7.6; B: The SEC-HPLC purity of Capto adhere loading condition optimization with pH 7.8. C: The SEC-HPLC purity of Capto adhere loading condition optimization with pH 8.0;

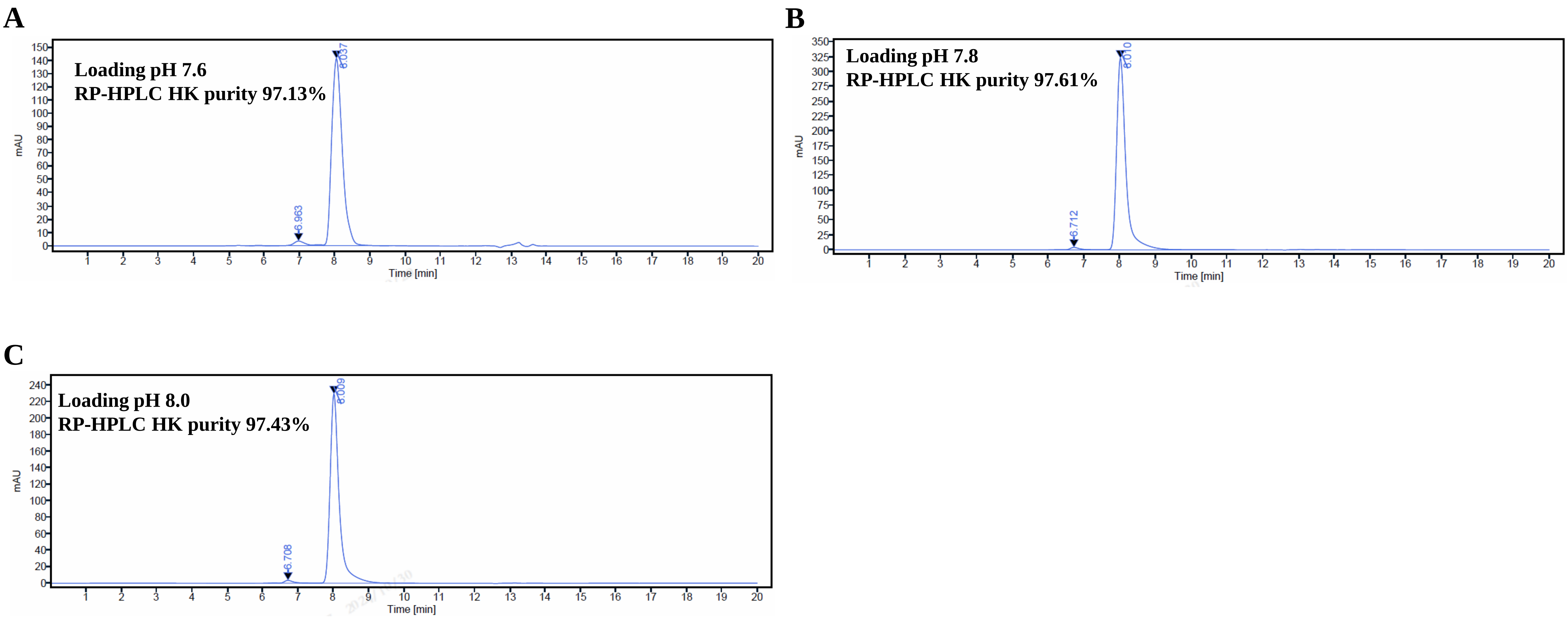

Supplementary Data 4: A : The RP-HPLC purity of Capto adhere loading condition optimization with pH 7.6; B: The RP-HPLC purity of Capto adhere loading condition optimization with pH 7.8. C: The RP-HPLC purity of Capto adhere loading condition optimization with pH 8.0;

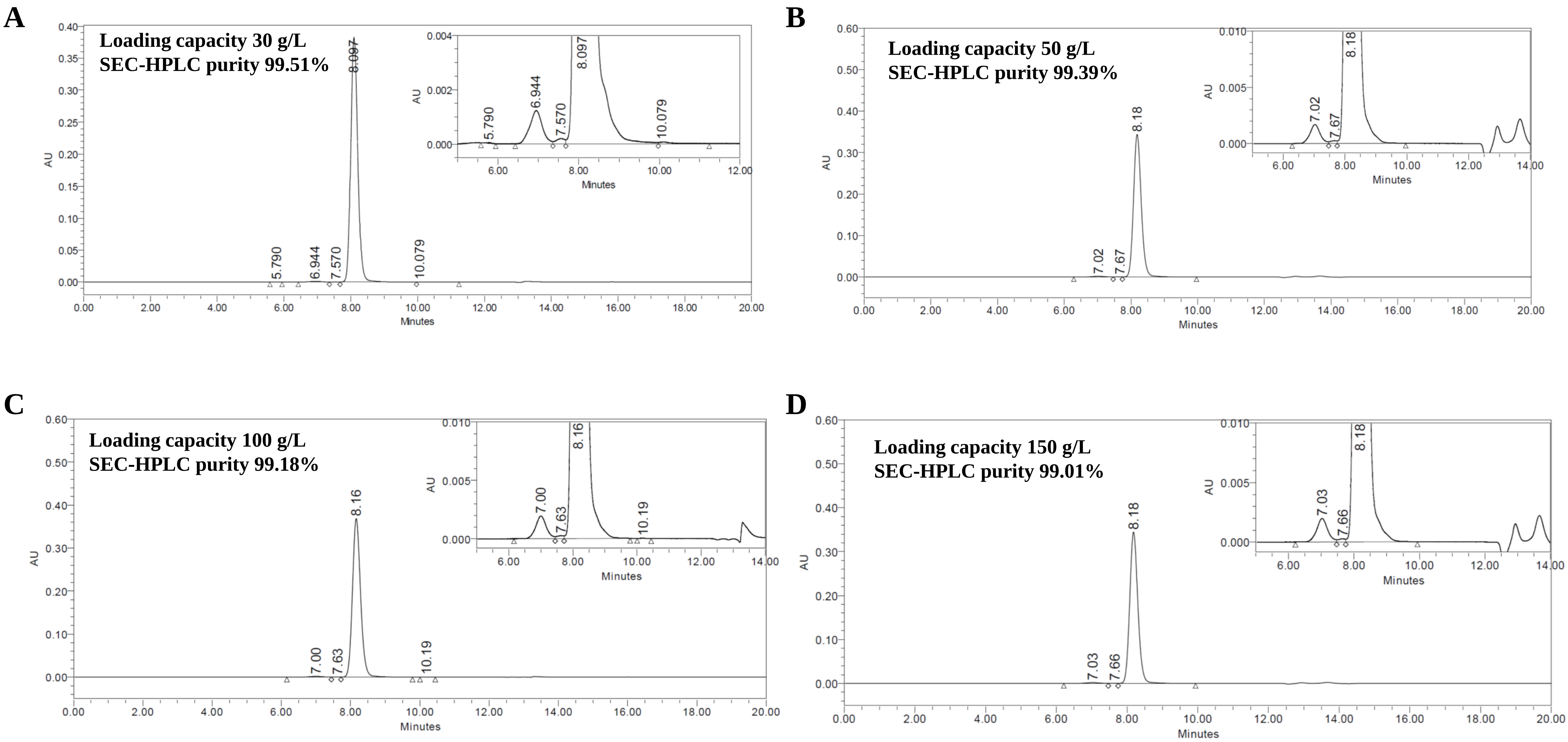

Supplementary Data 5: A : The SEC-HPLC purity of AEX capacity determination with 30 g/L; B: The SEC-HPLC purity of AEX capacity determination with 50 g/L; C: The SEC-HPLC purity of AEX capacity determination with 100 g/L; D: The SEC-HPLC purity of AEX capacity determination with 150 g/L.

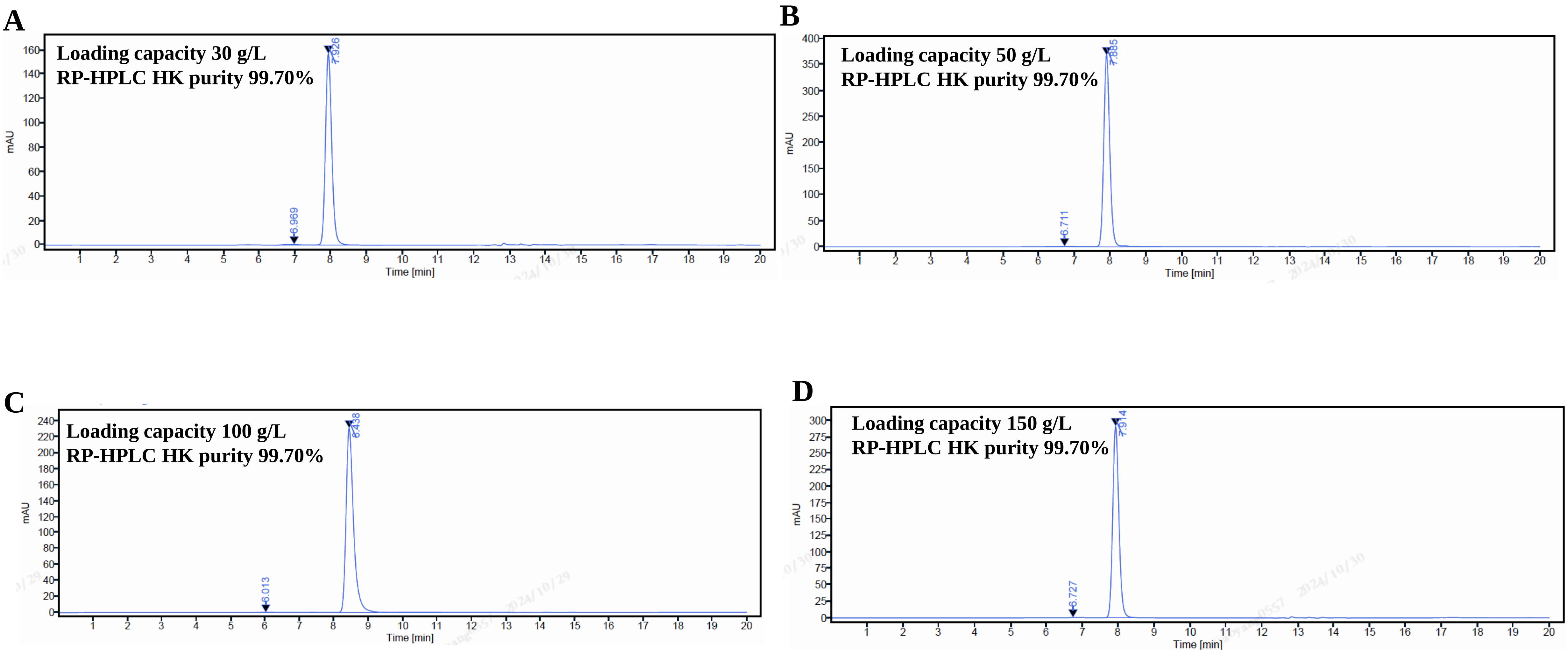

Supplementary Data 6: A : The RP-HPLC purity of AEX capacity determination with 30 g/L; B: The RP-HPLC purity of AEX capacity determination with 50 g/L; C: The RP-HPLC purity of AEX capacity determination with 100 g/L; D: The RP-HPLC purity of AEX capacity determination with 150 g/L.

A

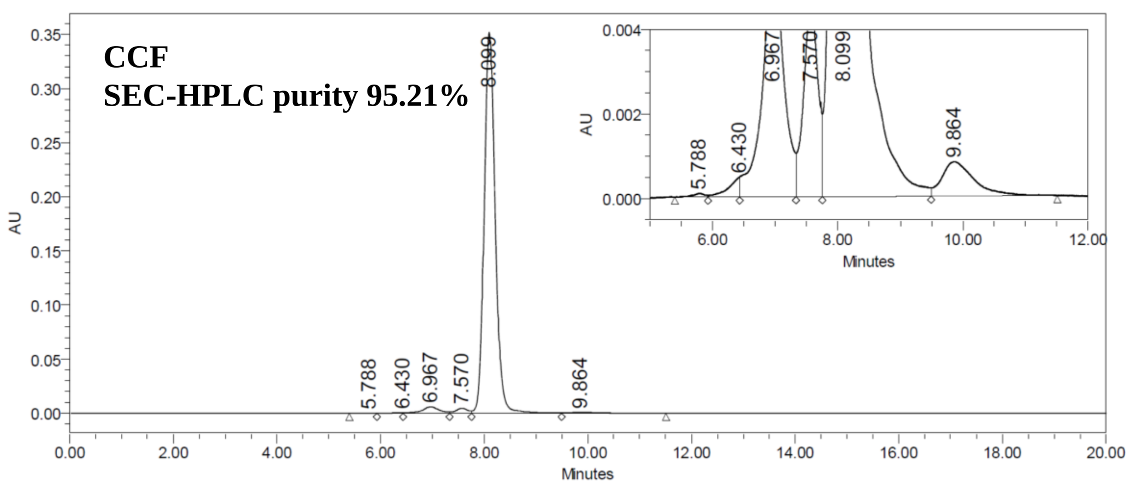

B

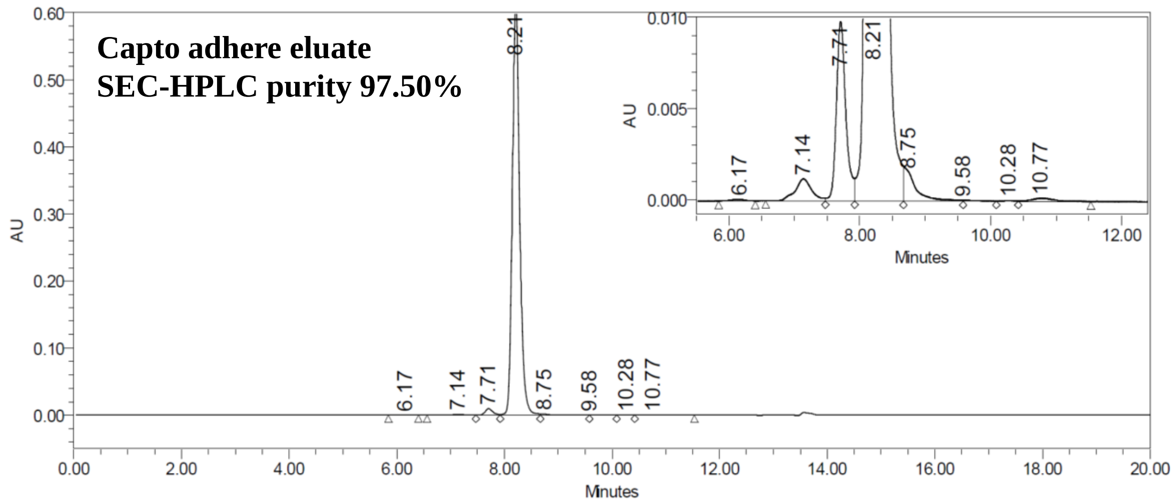

C

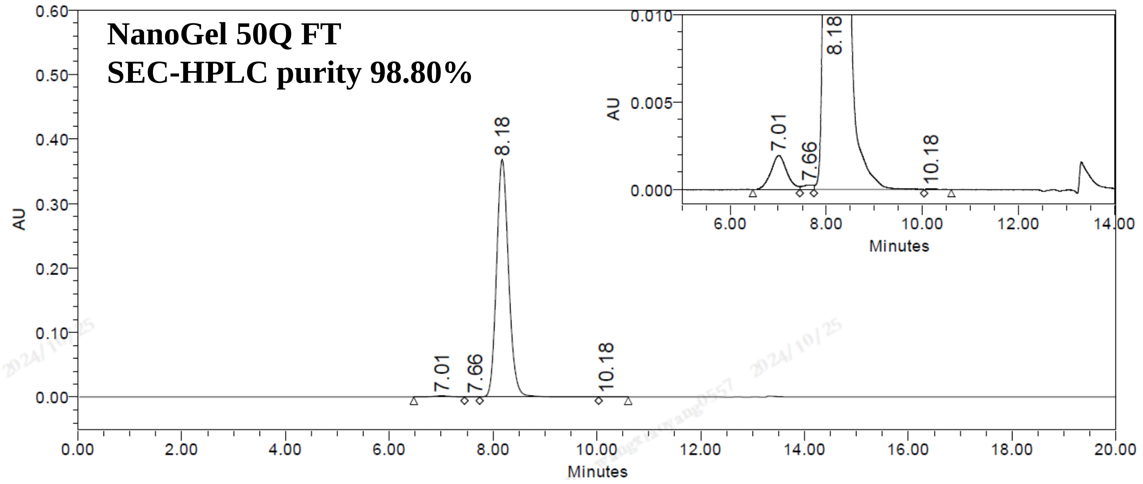

Supplementary Data 7: A : The SEC-HPLC purity of CCF; B: The SEC-HPLC purity of elution for Capto adhere; C: The SEC-HPLC purity of AEX flow-through.

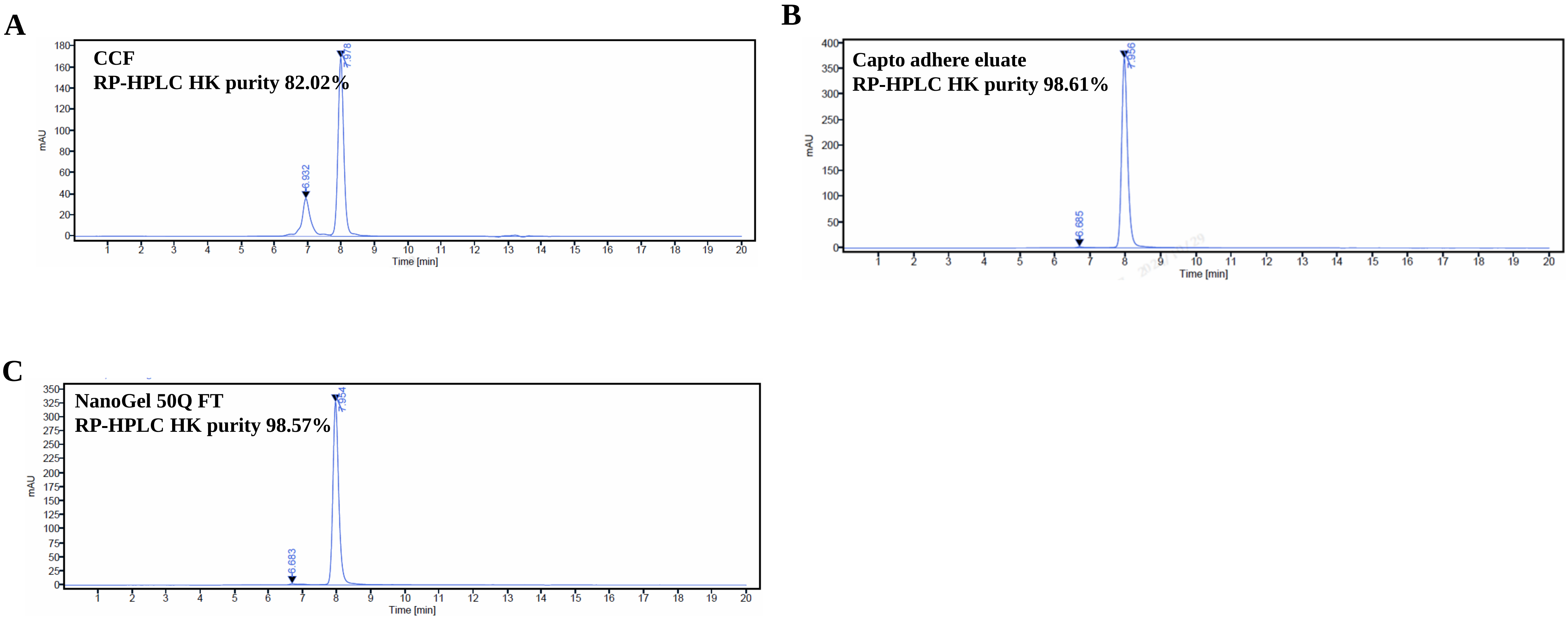

Supplementary Data 8: A : The RP-HPLC purity of CCF; B: The RP-HPLC purity of elution for Capto adhere; C: The RP-HPLC purity of AEX flow-through.
